# Peer Review of Grant Applications: Criteria Used and Qualitative Study of Reviewer Practices

**DOI:** 10.1371/journal.pone.0046054

**Published:** 2012-09-28

**Authors:** Hendy Abdoul, Christophe Perrey, Philippe Amiel, Florence Tubach, Serge Gottot, Isabelle Durand-Zaleski, Corinne Alberti

**Affiliations:** 1 AP-HP, Hôpital Robert Debré, Unité d’épidémiologie clinique, Paris, France; 2 Université Paris Diderot, Sorbonne Paris Cité, Paris, France; 3 INSERM, CIE 5, Paris, France; 4 Institut de cancérologie Gustave Roussy, Unité de recherche en sciences humaines et sociales, Villejuif, France; 5 AP-HP, Hôpital Bichat-Claude Bernard, Département d’épidémiologie, biostatistiques et recherche clinique, Paris, France; 6 AP-HP, Unité de recherche clinique en économie de la santé, Département de la recherche clinique et du développement, Paris, France; University of Michigan, United States of America

## Abstract

**Background:**

Peer review of grant applications has been criticized as lacking reliability. Studies showing poor agreement among reviewers supported this possibility but usually focused on reviewers’ scores and failed to investigate reasons for disagreement. Here, our goal was to determine how reviewers rate applications, by investigating reviewer practices and grant assessment criteria.

**Methods and Findings:**

We first collected and analyzed a convenience sample of French and international calls for proposals and assessment guidelines, from which we created an overall typology of assessment criteria comprising nine domains relevance to the call for proposals, usefulness, originality, innovativeness, methodology, feasibility, funding, ethical aspects, and writing of the grant application. We then performed a qualitative study of reviewer practices, particularly regarding the use of assessment criteria, among reviewers of the French Academic Hospital Research Grant Agencies (Programmes Hospitaliers de Recherche Clinique, PHRCs). Semi-structured interviews and observation sessions were conducted. Both the time spent assessing each grant application and the assessment methods varied across reviewers. The assessment criteria recommended by the PHRCs were listed by all reviewers as frequently evaluated and useful. However, use of the PHRC criteria was subjective and varied across reviewers. Some reviewers gave the same weight to each assessment criterion, whereas others considered originality to be the most important criterion (12/34), followed by methodology (10/34) and feasibility (4/34). Conceivably, this variability might adversely affect the reliability of the review process, and studies evaluating this hypothesis would be of interest.

**Conclusions:**

Variability across reviewers may result in mistrust among grant applicants about the review process. Consequently, ensuring transparency is of the utmost importance. Consistency in the review process could also be improved by providing common definitions for each assessment criterion and uniform requirements for grant application submissions. Further research is needed to assess the feasibility and acceptability of these measures.

## Introduction

Peer review is the most commonly used method for evaluating scientific research [1]. Peer review of manuscripts submitted for publication has been widely studied, and uniform requirements have been published to ensure transparency of the review process in this setting [2], [3]. In contrast, few studies have investigated peer review of grant applications, for which no international guidelines have been issued to date [4]. A recent study by the European Science Foundation highlighted differences in grant application review across countries and institutions [5]. In practice, grant applications are usually evaluated by internal and external reviewers, scored, and finally discussed by a review committee composed of the internal reviewers and funding organization members. The funding decision is based on the reviewers’ ratings and committee discussions.

Many aspects of the current grant application review process have been criticized. More specifically, lack of reliability has been strongly suggested based on studies showing poor agreement across ratings by external and/or internal reviewers [6]–[9]. Poor reliability might be interpreted by the scientific community as evidence of biases in the review process and therefore of unfair resource allocation [1]. Various methods have been suggested to improve the review process. For example, scoring could be replaced by other procedures such as the ranking method proposed by Hodgson et al. [10]; funding decisions could rely on the sandpit method, workshop review, or bibliometric data; or discretionary grants could be awarded [11]–[14].

Few studies have investigated the reasons for variations in assessments across reviewers of grant applications. A recent study investigated reviewers’ opinions of review procedures but did not collect data on the methods actually used by the reviewers [4]. The characteristics perceived by reviewers as indicating a good application were identified in one study [15] and the criteria used to assess clinical research questions in another [16]. The results of these studies point to reviewer subjectivity as a possible reason for the poor reliability of grant application review. Additional studies are needed to address this issue and to gain further insights into the methods used by reviewers to assess grant applications.

In a previous study [17], we investigated biases in the grant application review process used by the French Academic Hospital Research Grant Agencies (Programmes Hospitaliers de Recherche Clinique, PHRCs). The results showed that conflicts of interests affected the review process. Here, our goals were to identify the criteria used to assess grant applications and to determine how these criteria were applied by reviewers. Our study involved two steps: we first examined the review procedures used by French and international funding organizations and we then conducted a qualitative study to investigate the practices of PHRC reviewers.

## Materials and Methods

### Ethics Statement

The qualitative part of this study did not involve patients, and written consent was not required. Consent to participate was voluntary and was obtained by email. Anonymity and confidentiality of the interviews were guaranteed to all participants. An information sheet on the research objectives and confidentiality of study participation was read to each participant at the beginning of each interview. The participant was then asked to give oral consent and to allow audio recording of the interview. The institutional review board of the Paris North Hospitals, Paris 7 University, AP-HP, approved the study protocol, including the information sheet and oral consent procedure (N° IRB00006477).

### Survey of Procedures and Criteria used to Review Grant Applications

#### Sample of funding organizations

We constituted a convenience sample of French and international funding organizations. From each, we collected guidelines for reviewers and requirements for clinical research grant applications. French funding organizations were the seven regional PHRCs (Paris, North West, East, South West, Overseas, South Mediterranean, and Rhone-Alpes), the National PHRC, and the National Research Agency (Agence Nationale de Recherche, ANR). International funding organizations were the National Institutes of Health (NIH) in the US, Medical Research Council (MRC) in the UK, Canadian Institutes of Health Research (CIHR), National Health and Medical Research Council (NHRMC) in Australia, and European Science Foundation (ESF).

#### Data extraction and analysis

Grant review guidelines were provided directly to us from the seven French regional PHRCs; the South West regional PHRC used two sets of guidelines, one for methodologists and the other for nonmethodologists; and the guidelines were identical in the South West and Overseas PHRCs. Information on the other organizations was taken from the organization websites. One of us (HA) extracted information from the guidelines of each organization. We focused on assessment criteria and scoring methods. No data were collected on committee assessments or funding decisions. Two of us (HA and CP) analyzed the full set of criteria for each organization, identified the main assessment domains, listed the specific criteria used to assess these domains and recorded their frequency. Another of us (CA) validated the selection of the domains. The final list of domains and criteria was developed by consensus between CA, HA, and CP then validated by all authors.

### Qualitative Study on Reviewer Practices and Perceptions

#### Sample of reviewers

The sample was the same as in our previous qualitative study of PHRC grant application review [17]. Internal reviewers had reviewed applications submitted to the National PHRC and Paris Regional PHRC in 2008 or 2009; all eligible internal reviewers were asked to participate. External reviewers had been asked to review at least one grant application for the National or Paris Regional PHRC in 2009 and had reviewed at least one grant application in the last 3 years; they were selected by stratified randomization with the goal of obtaining a broad spectrum of views. Grant applicants were also selected by stratified randomization among the list of applicants who had submitted a proposal either for Paris Regional PHRC or the National PHRC in 2009. Stratification criteria were medical specialty and academic experience (i.e., junior vs. senior university-hospital physician), geographic location (Paris region versus rest of the country), type of stakeholder and, for applicants, rejection of a previous application.

Interviews were conducted until the saturation point was reached, i.e., until additional interviews produced no new information [18], [19]. In this type of study, the saturation point is usually reached after about 20 interviews.

#### Observation sessions

One of us (CP) attended the 2009 National and Paris Regional PHRC committee meetings (3 days for the national and 2 days for the regional meetings) to observe interactions and to make notes about the debates and reviewers’ attitudes. No audio recordings were obtained. These observation sessions provided direct information on the committee review process, as opposed to the rationalized reconstruction of events provided by post hoc interviews.

#### Interviews

We designed semi-structured interviews based on key themes identified from an analysis of the medical and sociological literature, French grant application review procedures, and official review documents. The main themes included in the final interview guide [17] were: career and reason for participating in the peer review process; review experience and experience with grant applications (for applicants); method used to review applications (for external and internal reviewers); difficulties in application assessment; perceived biases, strengths, and weaknesses of the review process; and ideas for improving the review process. When interviewees did not spontaneously bring up the review process, specific questions were asked to obtain information on the method used to review applications, use of assessment sheets, scoring of applications, perception of assessment criteria, and perception of the characteristics of a good application.

Each interviewee was invited by e-mail to participate in a study of the overall PHRC application review process. To minimize selection bias, no additional information about the study objective was given before enrollment. Consent to participate was obtained by e-mail. Anonymity and confidentiality of the interviews were guaranteed to all participants. Nonrespondents received an e-mail reminder every 2 weeks, up to a maximum of three reminders.

Interviews were conducted face-to-face at the participant’s workplace or by telephone by two of us (CP, a science sociologist; and HA, an epidemiologist trained in semi-structured interviewing by CP). Neutrality of the interviews was ensured by the fact that neither interviewer was involved in the grant application review process. The interviews were audiotaped and transcribed verbatim anonymously by an individual who was not otherwise involved in the study. Biographical information on each participant was collected at the beginning of each interview.

#### Analysis of interviews and observation sessions

The transcribed interviews were analyzed and coded by CP and HA, who combined case-oriented and variable-oriented strategies [18], [19]. Each interview was parsed by theme, and recurring themes were identified inferentially. Similarities and differences in thematic contents yielded variables across the cases. Data on observation sessions was explored by CP and HAL, following the same methodology, and compared with interview analyses [20]. The interviewers and another author (PA, sociologist) discussed the development of the themes and variables and validated the process. Cross-validation of the thematic analyses was undertaken at the same time by HA and CP using text analysis software (Tropes, Semantic Knowledge, Paris, France) [21]. The results of the analyses were compared and discussed among all authors. Interview patterns and differences between interviews or observation data were identified. Three main topics about grant review processes were identified: internal reviewer practices, external reviewer practices and the assessment process during the committee meetings. Quotes are given in the manuscript to illustrate the range of response. Interviewee characteristics are described in Appendix S1.

## Results

The results are reported according to RATS qualitative research review guidelines [22], [23].

### Survey of Procedures and Criteria used to Review Grant Applications

#### Overall description of grant application review procedures

Fourteen calls for proposals (five international and nine national) were investigated. All funding organizations used a two-step assessment process for all calls: the applications were first reviewed by internal and/or external reviewers then discussed by a committee. For three international calls, the review procedure included specific recommendations to take into account the applicant’s replies to reviewer comments during the assessment. Details on each procedure are provided in Tables 1 and 2. Additional information on French PHRC procedures have been reported previously [17].

**Table 1:** Guidelines for peer review of grant applications issued by the eight French Academic Hospital Research Grant Agencies.

	National PHRC	Paris PHRC	North West PHRC	East PHRC	West PHRC	South West PHRC (for methodologists)	South West PHRC (fornon- methodologist)	Rhone Alpes PHRC
***Scoring system***								
**Global scoring of the proposal**	Yes	Yes	Yes	Yes	Yes	Yes	Yes	Yes
Scoring method	Numerical	Alphabetical	Numerical	Numerical	Numerical	Numerical	Numerical	Alphabetical
Range of score	[4]–[12]	A, B, C,D, E	[5]–[20]	[0–20]	[5]–[20]	[14–56]	[7]–[28]	A+, A−, B, C, Other
Sum of individual criterion scores	Yes	No	Yes	Yes	Yes	Yes	Yes	No
Decision of funding based onproposals score	Yes	No	Yes [5]–[10]:reject; [11]–[15]discuss; [16]–[20]fund	Yes, [0–10]: reject	No	No	No	Yes, A+, excellent;A−, Good; B, Fair; C, Insufficient
***Individual scoring of each criterion***								
Scoring grid supplied	Yes	Yes	Yes	Yes	Yes	Yes	Yes	Yes
Scoring method	Numerical	Qualitative	Numerical	Numerical	Numerical	Numerical	Numerical	Alphabetical
Score ranking	[1]–[3]	Poor, Fair, Good, Excellent	[1]–[4]	[0–5]	[1]–[4]	[1]–[4], [2]–[8], [5]–[20], [4]–[16]	[1]–[4]	A+, A−, B, C, Other
Weighting of criteria	No	No	No	No	No	Yes	No	No
Sum of individual criterion or itemscores	No		No	No	No	Yes	No	Yes
	**National** **PHRC**	**Paris PHRC**	**North West PHRC**	**East PHRC**	**West PHRC**	**South West PHRC (for methodologists)**	**South West PHRC** **(for** **non- methodologist)**	**Rhone Alpes PHRC**
Guidelines about the scoring method	Yes, 1: theworse, 3: thebest		Yes 1,insufficient;2, fair; 3,good; 4,excellent	Yes 0, very inadequate; 1, inadequate, 2,open to criticism,3, acceptable;4,very good; 5, excellent	Yes 4, best possible score;1, worst possible score	Yes 1, worst possiblescore; 4, best possible score	Yes 1, worst possiblescore; 4, best possible score	Yes A+, excellent; A−, Good; B, Fair; C, Inadequate; Other, Out of scope of review
***Written report***	Mandatory	Mandatory	Mandatory	Mandatory	Mandatory	Optional	Mandatory	Optional
Structured report	No	No	No	No	No	No	Yes	No

**Table 2:** Guidelines for peer review of grant applications issued by one French national and five international funding organizations.

	ANR	NIH	MRC	ESF	CIHR	NHMR
***Peer review scoring system***						
**Global scoring of the proposal**	Yes	Yes	Yes	Yes	Yes	Yes
Scoring method	Alphabetical	Numerical	Numerical	Numerical or alphabetical	Numerical	Numerical
Score ranking	A, B, C	[1]–[9]	[1]–[10]	[[1]–[5] or A,B, C, D, E	[0–5]	[1]–[7]
Sum of individual criterion or item scores	Yes	No	No	Dependingof the call	No	Yes
Decision of funding based on proposals score	Yes, A : [26]–[30] Excellent,B [[21]–[25] Good, C:[0–20]not ready	Yes 1, exceptional;2, outstanding; 3, excellent; 4, very good; 5, good; 6, satisfactory; 7,fair; 8, marginal; 9, poor	Yes [1]–[2] unacceptable, [3]–[4] potentially useful,[5]–[8] good qualityresearch, [9]–[10]excellent quality research	No	Yes, [0.0–3.4]Not fundable,[3.5–4.9] Maybe funded	Yes, [4]–[7]: potentially fundable
***Individual scoring per criterion***						
Scoring grid supplied	No (guidance)	No (guidance)	No (guidance)	No (guidance)	No (guidance)	No (guidance)
Scoring method	Numerical	Numerical	No	Numerical or alphabetical	No	Numerical
Score ranking	[0–5]	[1]–[9]		[1]–[5] or A, B,C, D, E		[1]–[7]
Weighting of criteria	No	No		Dependingof the call		Yes
Sum of individual criterion or item scores	No	No		Dependingof the call		Yes
	**ANR**	**NIH**	**MRC**	**ESF**	**CIHR**	**NHMR**
Guidelines about the scoringmethod	Yes, 0: notaddressed or outof scope, 1: poor,2: borderline, 3: fair to good, 4:very good, 5: excellent	Yes, 1: exceptional,2: outstanding, 3: excellent, 4: very good, 5: good, 6: satisfactory, 7:fair, 8: marginal, 9: poor		No		Yes, [4]–[7] : potentially fundable
***Written report***	Mandatory	Mandatory	Mandatory	Mandatory	Mandatory	Mandatory
Structured report	Yes	Yes	Yes	Yes	Yes	Yes

#### Description of reviewer assessment practices

The assessment procedure included three parts: global scoring of the application, detailed assessment of specific criteria, and a written report. Global scoring was required for all 14 calls. The score was numerical for 10 calls, qualitative for 3 calls, and either numerical or qualitative for 1 call. Tables 1 and 2 show the scoring guidelines. An evaluation of specific criteria was required for all 14 calls, and the French PHRC guidelines involved completing a checklist of criteria. The criteria were scored in 12 of the 14 calls, using various methods and weighting procedures (Tables 1 and 2). For eight calls, the criteria assessment was used to compute the global score. A written report was required for 12 of the 14 calls, although guidelines about the structure of the report were provided for a single call.

#### Description of assessment criteria

Tables 3 and 4 list the criteria listed in the review guidelines for each call. The median number of criteria was five per call (range, 3–8). Table 5 reports our overall typology of assessment domains and criteria. We identified nine assessment domains.

**Table 3:** Criteria for grant application assessment recommended by French funding organizations.

	National PHRC	Paris PHRC	North West PHRC	East PHRC	West PHRC	SouthWest PHRC (formethodologists)	South WestPHRC (for non methodologists)	Rhone Alpes PHRC
**Number of criteria** **(n)**	4	7	5	5	7	5	8	4
**Details (weighting and score if applicable)**	Feasibility (3 points)	Relevance to the call (qualitative score)	Budget assessment (not scored)	Budget assessment(not scored)	Budget andpartnership (4 points)	Feasibility (16 points)	Adequacy of resources(4 points)	Feasibility (alphabetical score)
	Methodology (3 points)	Ethical and legal considerations (alphabetical score)	Ethical considerations(4 points)	Ethical considerations (not scored)	Ethics (bad, good,very good)	FinancialConsiderations(4 points)	Ethics (not scored)	General information (alphabetical score)
	Originality (3 points)	Feasibility(qualitative score)	Feasibility (4 points)	Feasibility (5 points)	Feasibility (4 points)	Methodology(20 points)	Feasibility(4 points)	Methodology (alphabetic score)
	Usefulness (3 points)	Financial considerations (qualitative score)	Methodology (4 points)	Methodology(4 points)	Methodology of the study (4 points)	Objectives and hypotheses (8 points)	Methodology (4 points)	Scientific rationale (alphabetical score)
		Methodology (qualitative score)	Originality (4 points)	Originality (5 points)	Originality andimpact (4 points)	Perspectives (8 points)	Originality(4 points)	
	**National PHRC**	**Paris PHRC**	**North West PHRC**	**East PHRC**	**West PHRC**	**South West PHRC** **(for methodologists)**	**South West** **PHRC (for non methodologists)**	**Rhone Alpes PHRC**
		Originality of the expected result (qualitative score)	Relevance of the subject(4 points)	Relevance of thesubject (5 points)	Quality and monitoring (bad, good, very good)		Prior publications(4 points)	
		Usefulness of the expected result (qualitative score)			Relevance (4 points)		Quality of writing(4 points)	
							Usefulness (4 points)	

**Table 4:** Criteria recommended for grant application assessment by one French and five international funding organizations.

	ANR	NIH	MRC	ESF	CIHR	NHMR
**Number of criteria (n)**	6	7	6	5	5	3
**Details (weighting and** **score if applicable)**	Global impact of the proposal (5 points)	Approach (9 points)	Ethics andresearch governance	Applicant(s) (numerical or alphabetical)	Applicant(s)	Scientific quality (50%)
	Mobilization ofresources (5 points)	Budget (not scored)	Importance	Budget (numerical or alphabetical)	Environment forthe research	Significance and innovation (25%)
	Project management(5 points)	Environment(9 points)	Justification of resources requested	Relevance and impact of the proposed research (numerical or alphabetical)	Impact for the research	Track record (25%)
	Quality of the consortium (5 points)	Innovation (9 points)	Other considerations (collections of human material, DNA, etc.)	Research environment (numerical or alphabetical)	Originality of the proposal	
	Relevance of the proposal to the call(5 points)	Investigator(s) (9 points)	Risks of research misuse	Scientific quality (numerical or alphabetical)	Research approach	
	Technical and scientific quality (5 points)	Other criteria according to thescope ofthe grant	Scientificpotential (environment, research plans)			
		Significance (9 points)				

**Table 5:** Typology of assessment criteria derived from our sample of calls for proposals.

Domains	Number of calls (N = 14)
**Domain 1: Relevance of the project to the call**	**3**
Duration of the project	1
Relevance of the proposal to the scope of the call	2
Relevance of the applicant’s characteristics to the scope of the call	2
**Domain 2: Scientific interest**	**14**
Usefulness of the research for patients	9
Usefulness of the research for clinical practice	8
Relevance of the question and study concept to improving public health or scientific knowledge	11
Literature review on the scientific and medical context	5
Existence of preliminary studies	2
Need for research in this area	1
Existence of competing studies	1
**Domain 3: Originality and impact of the research**	**13**
Potential for providing original data not available in the literature	3
Potential for changing medical practice	1
Potential for international publications	1
Robust plan for disseminating the results	3
Potential fundamental outcomes in the science and/or practice of clinical medicine or public health or fundamental changes in health policy	2
Potential creation of new knowledge	1
Potential change of the concepts, methods, technologies, treatments, services, or preventive interventions that drive this field	1
Potential for opening new scientific and technical perspectives	4
Potential new understanding of a topic	1
**Domain 4: Innovation**	**6**
Proposal innovative and original with respect to the technical and scientific aspects	5
Use or improvement of novel theoretical concepts, approaches, or methodologies, instrumentation or interventions	1
Potential for creation of new ideas or direction of research	1
**Domain 5: Methodology**	**14**
Clarity of the objectives of the study	5
Clarity of the rationale	1
Adequacy of the design and methods to the objective	8
Relevance of inclusion criteria	5
Relevance of noninclusion criteria	5
Relevance of randomization methods	2
Sample size estimation	4
Definition of stopping rules	2
Relevance of assessment criteria	6
Quality of data-management	2
Relevance of statistical analysis	5
Quality of pharmaceutical manufactures	1
Methodological quality of the study design	3
Competitiveness of the study design	1
**Domain 6: Feasibility**	**14**
***Proposal***	
Recruitment feasibility	5
Description of the acts and sequences of the research	2
Evaluation of risks and problems of feasibility	3
Realistic time frame	6
Description of the management of adverse events	2
Description of pharmaceutical considerations	3
Description of data management methods	2
Quality of budget assessment	1
Feasibility of the approach	3
Involvement of industry if needed	1
Control of methods employed in the proposal	2
Mobilization of human and logistic resources	4
No competitive proposals	1
In case of proof of concept research, feasibility of further research projects	1
***Applicants***	
Quality of collaborations	10
Complementarity of collaborations	8
Prior publications of the applicant	4
Track record of the applicant	2
Relevance of the applicant (training and experience)	2
Relevance of the co-investigators (training and experience)	1
Relevance of the research field in the work of the applicant	4
***Environment***	
Adequacy of the research location	4
Adequacy of the team and the research environment	4
Involvement in the field of the research	2
Mobility and career development aspects	1
**Domain 7: Financial considerations**	**12**
Calendar and schedule of the research	2
Time requested adequate	1
Description of supplementary funds	2
Description of resource allocations	4
Adequacy of resources	6
Description of collaborations	1
Good value for money in terms of the resources being requested	1
**Domain 8: Ethical considerations**	**6**
Assessment of the patient benefit-risk ratio	2
Description of patient consent forms and information	2
Description of patient follow-up	1
Ethical review and research governance clear and acceptable	1
Risk of research misuse addressed	1
**Domain 9: Quality of writing**	**2**
Global quality of writing	1
Arrangements for promotion of the public understanding of science	1

- Relevance of the research project to the call for proposals, amount of funding requested, and characteristics of the applicant. Information on this domain was required by only three funding organizations.
- Scientific relevance of the research project or study question. Information on this domain was required for all 14 calls. Reviewers were asked to broadly assess the usefulness of the research project or to provide specific information on previous studies in the field and on the literature review supplied by the applicant.
- Originality was to be assessed for 13 of the 14 calls, based on the potential impact of the research project as assessed by the reviewer, in particular based on the potential for publication.
- Innovativeness referred to the technological, technical, or methodological innovations used or investigated in the research project. Information on this domain was required for only 6 calls. The innovativeness domain was sometimes included in the originality domain.
- Methodology was a domain on which information was required for all 14 calls. In weighted scoring systems, a high weight was given to this domain. The review guidelines included specific questions about numerous methodological issues such as sample size estimation and quality of the study design (Table 5).
- Feasibility. This domain encompassed a number of issues pertaining to the research project, characteristics of the applicant (e.g., previous publications and collaborations), and scientific context (e.g., competing research projects). In the guidelines for some of the calls, the feasibility domain included methodological issues (e.g., required sample size), adequacy of the requested funds, and ethical aspects (e.g., about patient consent to participation) (Table 5).
- Financial considerations and requested funds. This domain included specific questions on the planning of the project and description of necessary resources. Scoring was not always required.
- Ethical considerations, including potential risks to patients. In some cases, this domain included methodological issues such as the management of missing data or of patients lost to follow-up (Table 5). It was often assessed qualitatively, as opposed to scored (Tables 1 and 2).
- Writing or readability of the application. Only two calls requested information on this domain, which was usually assessed subjectively. The guidelines for one call included a question on how well the application could be understood by nonscientists (Table 5).

### Qualitative Study on Reviewer Practices and Perceptions

#### Characteristics of the interviewees

We invited 128 reviewers (45 internal and 83 external reviewers), of whom 76 (40 internal and 36 external reviewers) accepted to be interviewed and 65 accepted to participate in the qualitative study; 11 reviewers were interviewed and consented to the study but finally were not available for the study interviews. The interviews began after the committee meetings, in June 2009, and ended in November 2010. Thirty-six (37%) interviews were conducted by telephone. Two interviewees refused to be recorded during the interview, and two recordings were of insufficient quality to allow transcription; the written notes taken during the interviews allowed us to use these four interviews. The saturation point was reached after 38 interviews of internal reviewers and 27 of external reviewers. Table 6 reports the main characteristics of the 65 participants. Interview length ranged from 15 to 91 minutes (median, 31 minutes). Most participants were pleased to participate and to discuss the grant application review process. The main reason for refusing to participate was lack of time.

**Table 6:** Characteristics of the 65 reviewers who participated in the qualitative study.

Characteristics	N	N (%)	Internal reviewers (n = 38)	External reviewersc (n = 27)
**Age (years)**	59			
30–39		1 (1)	0	1
40–49		27 (46)	18	9
50–59		24 (41)	10	14
60–69		7 (12)	5	2
**Sex**				
Male		48 (74)	29	19
Female		17 (26)	9	8
**Geographic area**				
Paris area		45 (69)	31	14
Rest of the country		20 (31)	7	13
**Specialty**				
Medicine		37 (32)	14	7
Surgery		5 (8)	2	3
Methodology		10 (15)	9	1
Psychiatry		3 (5)	1	2
Obstetrics and gynecology		2 (3)	1	1
Biology		17 (26)	8	9
Anesthesia		5 (8)	2	3
Other		2 (3)	1	1
**Medical and academic status**				
Senior academic doctor		60 (92)	37	23
Junior academic doctor		0 (0)	0	0
Non-academic doctor		5 (8)	1	4
**Experience in grant review (years)**	**65**			
0–2		13 (20)	11	2
3–5		21 (32)	9	12
>5		19 (29)	6	13
Unknown		12 (19)	12	0

Three themes emerged from our analysis and are detailed below: practices of external reviewers, practices of internal reviewers and the assessment process during the committee meetings.

#### Practices of external reviewers

We evaluated the practices of external reviewers based on time spent on the review, whether reviewers looked at previously published studies, whether reviewers used funding organization checklists, and writing of the report.

### • Time Spent on Reviewing Grant Applications

The interviews showed wide variations in the time spent reviewing applications, from a few hours to several days: *“We read a little … I would say … adding it all up … it must take a good ten hours I think. […] And I will not spend more than ten hours – I can’t anyway!*” (external reviewer 17) and *“It depends on the project, but one or two days*” (external reviewer 15). Most reviewers spent several work sessions on each application: *“I take notes as I go along, I often need time, well I don’t know what a decent time would be […], but sometimes I spend quite some time … I read through the application once, to get an overall idea of project, its goals and approach, the methodology, and then I read it a second time more carefully, and I usually make a few comments. So first I try to get a broad picture of the research project and then I focus on the details […]*” (external reviewer 12). More rarely, reviewers processed each application in a single session: *“When I have enough time, I focus and I read the entire application in one session – so I arrange to have enough time, an afternoon, or whatever time I need to read the application, and as I read I make notes.*” (external reviewer 10). Some reviewers also complained about lack of time and short deadlines: *“The deadlines are always very short; when you apply for a grant, you always find that getting the answer takes a very long time … […] but for me … every time I’m given only ten days to send in my report!”* (external reviewer 26). However, a few reviewers felt that time was not a problem because they always reviewed the applications at the last minute: *“Anyway, it’s true that the deadline is always too short, but you know, even if we received the applications one month before the deadline, we would wait until the last minute to review them, or at least I would (smile).”* (external reviewer 10). Finally, some reviewers felt that the ability to meet the deadline was chiefly dependent on reviewer behaviors: *“It’s not a real concern; there are people who put in the work and people who don’t. Some people miss the deadline regardless of the circumstances.”*(external reviewer 8).

### • Looking at Previously Published Studies

Reviewers varied in their practices regarding referral to previously published studies. Most external reviewers performed a literature search, mainly to assess the scientific relevance of the proposal: *“I read the proposal and, when I have time, I read the literature, at least… I always try to take a quick look at the literature to assess the relevance [of the proposal]…”* (external reviewer 25). Some reviewers searched the literature only on a case-by-case basis, to confirm an opinion or to explore specific issues: *“I rarely search for articles. Except on matters that puzzle me, or if I feel the proposal is incomplete – then, I write a note and I check on PubMed to see whether it is correct it is correct… But not routinely, I must admit.”* (external reviewer 24).

### • Use of the Assessment Checklists Recommended by the Funding Organizations

For the 2009 national and regional PHRCs, assessment checklists were provided to all reviewers as an aid to reviewing and scoring the applications. Most of the external reviewers found these checklists helpful: *“The checklists clarify the way in which we see the project. They help us become aware that our approach to assessing projects is sometimes a bit fuzzy. They give us a clearer picture of the overall project”* (external reviewer 16). The checklists were also perceived as providing information on the points that were important to the funding organization: *“The checklists help us to understand the committee’s point of view … the hierarchy of the assessment parameters, and they are important to help us determine how to write the final report.”* (external reviewer 16). The reviewers felt the checklists might help them write their own applications in the future: *“I wrote a proposal just before phoning you. The checklist is very helpful because we can find out right away what is missing…”* (external reviewer 20). However, some reviewers felt the checklists were difficult to complete: *“It is not always easy, is it? […] Some of the items may not be relevant to an individual proposal and are therefore difficult to answer. When I review several proposals, it is obvious to me that there are differences in the usefulness of the checklist, depending on the specific features of each proposal. In general, the checklist is not too difficult to complete.”* (external reviewer 11). Another criticism related to the broad nature of the assessment criteria: *“The items are good, but I think that for each item there should be a list of sub-items and response options. For example, for assessing the methodology, in the checklist that was given to me, the item was just “methodology”. The reviewer has to provide details on the method chosen, its appropriateness to the study question, whether the nature of the data allows the statistical analysis, whether the statistical methods chosen are appropriate, and whether the sample size is large enough.”* (external reviewer 15). A few reviewers strongly criticized the scoring of proposals: *“You can give scores from 0 to 20, it’s the same thing, there will be scores of 18, 12, 4 […] It makes no sense! First, because we have no control over the quality of the reviewers or their scoring practices. I am not even sure that all the reviewers read the long list of explanations on the scoring procedure. They don’t even read it. So it is useless. Now, it makes everyone happy … and it rationalizes rejections: “Here, you see, you got a bad score, so we will not not fund [your proposal]”* (external reviewer 8). In practice, most of the reviewers completed the checklists at the end of the review process, as a means of supporting rather than of developing their opinion: *“I always used the checklists at the end […]. I formed my own opinion of the proposal, by making a critical appraisal of the proposal on my own, and when that was done I matched my comments to the checklist.”* (external reviewer 22). Nevertheless, reviewers felt the checklists served a purpose: *“It has never happened to me that, after having reviewing each point of the proposal, my final score was very different from what I expected. […]. I think the checklists are useful – clearly, they can be very useful when the proposal is rejected and returned to the applicant […], and also for helping to rank proposals.”* (external reviewer 9).

### • Writing the Final Report

Few details were given about the writing of the final report. One reviewer felt that the report should only provide a scientific opinion, without assessing whether the proposal should be funded: *“I don’t think it it is the case for French PHRCs, but sometimes other organizations ask us to make the final decision about funding, and I don’t think this is an appropriate request to make to external reviewers, […] who have not seen all the proposals and consequently cannot rank them.”* (external reviewer 16). Most reviewers felt that their report should be designed to help the applicants improve their proposals: *“If our report only says “oh! your proposal is bad”, that’s not interesting, not constructive, not useful.”* (external reviewer 10); and *“In my opinion, one of the most important aspects of the peer review is the opportunity to improve [the proposal].”* (external reviewer 12). Thus, the review process was sometimes perceived as a way to help rather than to judge the applicants: *“less like a judgment and more like help”* (external reviewer 18).

### Practices of Internal Reviewers

We assessed three components of the practices of internal reviewers: the material conditions of the reviews- in particular, time spent and literature search-, the use of assessment checklists, and the use of external reviewer reports.

### • Material Conditions of the Reviews

The time spent on each application varied less among internal than among external reviewers. Internal reviewers usually spent 1 to 2 hours on each application: *“I think that now I spend one hour …no, two hours. One hour the first time I read it, then one hour to read it again, so two full hours.”* (internal reviewer 33). The time spent on each application was perceived by the internal reviewers as dependent on the quality of the external reviewers’ assessments, on whether the external reviewers met the deadline, on the number of external reviewers, on the existence of disagreements among external reviewers regarding the application, and on the level of expertise of the internal reviewer in the field relevant to the application. Most internal reviewers did not perform routine literature searches, instead using previously published data only to support the opinion they had already formed (*“We read the proposal and we check the references if necessary. We do not check whether they exist or not, but we check them if we disagree or if we believe that new data have been published.”*, internal reviewer 1) or to assess the applicant’s reputation and ability to publish (*“I check the publications on Medline or in the proposal, and I see if the applicant has been able to produce papers that were sound.”,* internal reviewer 10).

### • Use of the Assessment Checklists Recommended by the Funding Organizations

Most of the internal reviewers had opinions similar to those of the external reviewers regarding assessment checklists. Thus, checklists were usually perceived as helpful, although a few internal reviewers criticized the scoring method: *“[…] I distrust numbers: you know that book on statistics that says “There are three kinds of lies, lies, damned lies, and statistics”! We can make the numbers say what we want them to say.”* (internal reviewer 18); and *“Summing to get a global score does not provide a global opinion – this point has been convincingly demonstrated. In general, the opposite happens and there is a “halo effect”. In general, reviewers form an overall opinion about the proposal and then they assign the scores and subscores based on that opinion.”* (internal reviewer 16).

### • Use of External Reviewers’ Reports

The internal reviewers relied heavily on the reports by the external reviewers. Some internal reviewers read the external reviewer reports before reading the application: *“ I take the report that is on the top of the stack and, since my role as an internal reviewer is that of a rapporteur, I read the experts’ reports before reading the proposal. So I read the two or three reports that I have. Then I form an opinion, since my job is to create a synthetic overview of the reports – I form a global opinion of the external reviewers’ perceptions and of the differences that may exist among them.”* (internal reviewer 18). Other internal reviewers read the applications first: *“As the internal reviewer, I read the proposal first to form my own opinion, then I read the two external reviewer reports; if they support my opinion, I don’t have much more work to do if I believe the reports are correct; if the two reports differ widely, I go into the details of the proposal; and if the reports do not support my opinion, I determine who is right, and sometimes I realize I had missed something.”* (internal reviewer 17). The quality of the external reviewer reports was perceived as crucial by the internal reviewers, who gave great importance to point-by-point analysis and discussion: *“When an expert writes ‘Excellent project that must be funded’ with a four-line assessment, the report goes straight to the wastebasket – it is not useful at all. A review is useful only if it analyzes and discusses each of the important points relevant to the funding decision. ”* (internal reviewer 20).

We identified various strategies used by internal reviewers to write their reports. Some internal reviewers wrote a synthetic overview of the external reviewer reports, usually without giving their own opinion: *“ I always try to restate what the external reviewers wrote, because I do not want to act as an ‘additional reviewer’ giving an opinion that would prevail over the opinions of others.”* (internal reviewer 19); and *“The job of the external reviewers is to give their opinion, whereas the internal reviewers act as rapporteurs, whose job is to assess whether these opinions are… founded or not, subjective or not… and whether their impact is limited or major.”* (internal reviewer 5). The internal reviewers sometimes sought to compare the detailed analysis in the external reviewer report with the score assigned by the external reviewer: *“When the report provides a detailed analysis, I try to look at each point to see whether I agree with the external reviewer […] and whether there is a discrepancy between the analysis and the score. […]. Scoring is relative, and my job is to try to find a balance.”* (internal reviewer 20). When external reviews were lacking or of poor quality, the internal reviewers sometimes acted as external reviewers. Furthermore, some internal reviewers sought to reconcile differences between external reviewer reports: *“Sometimes, when there were discrepancies, I had to make a choice.”* (internal reviewer 26). Internal reviewers who were thoroughly conversant with the relevant field sometimes gave precedence to their own opinions, rather than to those of the external reviewers: *“When I feel the field is one in which I have considerable expertise, I put my score in the final report, and I discuss the external reviewers’ opinions based on my interpretation – so I answer the concerns raised by the reviewers. […] So in this situation I act as a ‘super reviewer’”* (internal reviewer 14).

### Actual Perceptions and use of Assessment Criteria by Internal and External Reviewers

When the reviewers were asked about the criteria they used to assess proposals, most of them said they used the criteria in the national and regional PHRC checklists: scientific relevance of the proposal, originality of the study, methodology, feasibility, ethics, and financial considerations. Many reviewers felt that one or a few items were particularly important, whereas a few of them placed all the criteria on the same level: *“The report is only useful if it contains a detailed analysis of all the important points relevant to the funding decision.”* (internal reviewer 20). Table 7 lists the perceptions of criteria by internal and external reviewers.

**Table 7:** Reviewers’ perceptions about assessment criteria.

Perceptions	All reviewers (N = 65)	Internal reviewers (n = 38)	External reviewers (n = 27)
**Importance of assessment criteria**			
Every criterion is important in the assessment process	12	5	7
No concern about the importance of assessment criteria	19	17	2
One or several assessment criteria are important for the review	34	17	18
**Most important assessment criterion according to the participants**			
Originality	12	5	7
Methodology	10	5	5
Scientific interest	5	3	2
Feasibility	4	1	3
Originality and scientific interest of identical importance	1	0	1
Methodology and feasibility of identical importance	1	1	0
Depends on the proposal	1	1	0

- Originality of the study was perceived as the most important criterion (Table 7): *“Good projects are based on original ideas. […] If the proposal offers a sound rationale, and says ‘this is what we know, this is the current state of science in this field, and there are absolutely no data about this point, so this is the point we will investigate’ […]”* (internal reviewer 7). Although originality was rarely defined by the reviewers, some reviewers felt it was relevant to the potential impact of future publications: *“My main interest is in the originality and usefulness of the study – that is, in its scientific originality […] – the results the study will provide, that is the main point in my opinion.”* (external reviewer 10). Many reviewers perceived originality as deserving priority, despite the risk involved, as opposed to more pragmatic considerations such as feasibility: *“I feel that originality is very important. […] In my opinion, feasibility is less important than originality for a research proposal. […] When there is a flawed but very original idea, this idea can then be refined and improved as the process of research unfolds.”* (external reviewer 16).
- Methodology was the second most important criterion according to the reviewers. However, a few reviewers pointed out that advice from the reviewers can result in improvements in the methodology and that, consequently, applications should not be rejected based on methodology alone: “[…] *the methodology can be improved, and [what really matters] remains* the *relevance of the project itself.”* (internal reviewer 25).
- Scientific relevance was ranked third in importance among assessment criteria. However, most reviewers did not explain in detail how they decided that a study question was scientifically relevant. This criterion seemed to be perceived as reflecting the usefulness of the proposal for the scientific community or for patients: *“Does the proposal address a real issue? […] what is its relevance to patient management?”* (external reviewer 15).
- Feasibility was an important criterion for many reviewers: *“Clearly, you can have the best idea in the world, if you have only one-tenth of the patients needed to investigate it, there is no point in carrying out the project*. *Especially when it comes to funding, the money would be wasted.”* (external reviewer 9). However, most of the reviewers felt that feasibility was difficult to assess, either because the necessary information was not available (*“Well for feasibility, there is no doubt that we often lack the necessary information. We would have to know whether the applicant’s research group has other projects that compete with this one, the size of the population, the size of the group, and we don’t have information on any of these points.”*, external reviewer 25) or because the reviewers felt they lacked the necessary expertise (*“I am not capable of assessing feasibility. If someone tells me that 200 marijuana addicts are needed for an upcoming trial, then how can I know whether obtaining that number is feasible?”* (internal reviewer 1). We identified several strategies used in practice by the reviewers to assess feasibility. One of these strategies consisted in relying on personal experience: *“I think this assessment relies mostly on personal experience and on our knowledge about the topic…”* (external reviewer 12). Another strategy involved considering the reputation of the applicant and his or her scientific environment: *“What matters regarding feasibility is the applicant’s reputation, not practical feasibility. If an applicant previously conducted a project to term then submits another project, then this new project will probably also be completed. Feasibility is based on the individual, not on practical considerations.”* (internal reviewer 3); and *“In my opinion, a project does not come out of thin air! The project is developed within a research group that knows how to do a number of things … it’s not a castle in the air! … […] So we need to know … I don’t know, it is like when you buy a car, you are more confident if you buy it from someone whose car you know is reliable…”* (external reviewer 23). To assess reputation, the reviewers relied on their personal acquaintances with the applicants (*“I take a look at the team, and in general I know them so I know if they are able to do it or not”*, internal reviewer 10) or on previous publications by the applicants (*“They have already carried out projects, so they will be able to carry out this one. If they haven’t completed any projects, then they won’t be able to complete a new project. […]. It is always the same logic. If you are a researcher, you must publish.”*, external reviewer 18). Third, reviewers sometimes assessed feasibility based on expectations regarding patient enrollment. However, assessment of this point was perceived as very difficult in some cases: *“It can be difficult to predict […]. It is impossible to know how much energy the research team will put into enrolling patients, or how the study will be managed.” (internal reviewer 2). Reviewers had to check the sample size estimations and the recruitment rates: “They have to prove to us that they can enroll the necessary number of patients, for instance by stating that they see X cases each year, and given the inclusion and exclusion criteria, we therefore expect to enroll x% of those patients […]”* (internal reviewer 19). The study methodology and, more specifically, the sample size and patient recruitment issues were considered relevant to the feasibility assessment: *“In clinical research*, *the sinews of war are the patients. So there has to be a sample size estimation … then proof that the available number of patients will be sufficient to reach that size*. If *the number of patients is inadequate, the study will never be done; there is a famous law*, *I forget its name, saying* that *when you expect to recruit 100 patients then you actually enroll only 50, with difficulty: it’s always half the expected number.*” (internal reviewer 11).
- Ethical aspects were rarely viewed as important by the reviewers. The ethical acceptability of the study was often perceived as easily assessed and not critical for the review: *“I rarely encounter major ethical dilemmas and I am not sure that the reviewers are the best people from whom to seek advice on this point. If ethical obstacles exist, then that may have a very small impact [on the assessment], but I am not sure that the ethics of the project should be given the same weight as the other criteria or that ethical aspects should be assessed by the reviewers”* (external reviewer 14).
- Financial considerations were considered very important by many reviewers: *“[…] If the funding requirements are properly described, then they know how to manage a PHRC grant.”* (external reviewer 8). However, most reviewers felt that the appropriateness of the funds requested was too difficult to assess: *“We don’t know how to evaluate this. I am always very puzzled about this point. It’s guesswork, isn’t it? We look at things and I think that we don’t have the necessary training, or maybe we should have points of reference […].”* (external reviewer 4). Some reviewers felt they lacked the necessary skills to assess financial issues: *“I am not competent to give advice about the budget. […] This is not my area of expertise. I prefer to assess scientific issues […]. I often write that for this part of the assessment, I don’t know.”* (external reviewer 10).
- Finally, the quality of the writing was rarely mentioned by the reviewers, probably because quality was considered good overall:*“[Bad proposals] are very few and their number is decreasing over time, because quality is improving gradually.”* (internal reviewer 30).

### The Assessment Process during the Committee Meetings

The assessment process of the PHRCs committee meetings was explored during the observation sessions: during the committee meeting, each internal reviewer summarized the application and subsequently the reviewers’ reports then finally gave his or her own assessment. The committee then discussed the funding decision. All committee members participated in the discussion and had the opportunity to ask questions of the internal reviewers. In practice, the main assessment criteria discussed during the committee meetings were those on the PHRC assessment checklists: methodology, originality, and relevance. Financial considerations were discussed when the funds requested were felt to represent an excessively large percentage of the total funds available for the call. Feasibility issues were also explored, in particular based on the applicants’ résumés and previous applications. As the time available for discussing each proposal was short, the internal reviewer reports and the articulateness of the internal reviewers had a substantial impact on the discussions. Internal reviewers who delivered clear and well-reasoned presentations usually had their opinions accepted by the committee. In contrast, a more lengthy discussion was likely to unfold in response to presentations delivered in a hesitant manner or marked by inconsistencies. Finally, the funding decision was made by developing a consensus and not by majority vote.

## Discussion

### Main Findings

In the first part of this study, we identified review processes and criteria recommended by French and international funding organizations to assess grant applications. Considerable similarity in these processes and criteria was noted across calls for proposals. The main differences involved scoring methods, criteria weighting, and detailed definitions of criteria. We developed a typology of assessment domains that might prove useful for building common international guidelines about grant application review.

The second part of our study focused on French PHRCs and collected qualitative data on reviewers’ practices and perceptions. Both external and internal reviewers differed in terms of time spent on each proposal, referral to previously published data, scoring, and report writing. External reviewers perceived their role to be that of scientific experts or participants in the funding decision. In contrast, internal reviewers felt they should establish a consensus, perform an additional assessment of proposals, or act as “super reviewers”. Although most of the assessment criteria were accepted by all reviewers, opinions differed about the processes for scoring and committee decisions. On the one hand, internal and external reviewers had their own interpretations and weighting systems for the criteria, whereas on the other the consensus achieved during the committee meetings relied on a small number of criteria defined only very briefly. This discrepancy between the uniform requirements of funding organizations and the heterogeneity of reviewer practices may limit the reliability of assessment process and impair its ability to select the best applications.

### Strength and Weaknesses of Our Study

To our knowledge, this is the first study that simultaneously investigated funding organization requirements, assessment criteria, and reviewer practices. Previous studies focused either on assessment procedures [4], [5] or on grant application review methods [15], [16]. We used a convenience sample of French and international calls for proposals. Our objective was not to be exhaustive but to evaluate review procedures and to establish a typology of assessment criteria used by multiple funding organizations. We studied only 14 funding organizations, and our results should be compared to those obtained with other organizations. Furthermore, our qualitative study included only reviewers working with the French PHRCs. We chose a qualitative design to investigate reviewers’ perceptions without influencing their answers. Our goal was not to quantify or to obtain an exhaustive description of reviewers’ perceptions. Instead, we sought to improve our knowledge of review practices. The reviewers participated on a volunteer basis, and representativeness was achieved via stratified randomization. We checked the reliability of our results by triangulation (i.e., observational sessions, interviews, and text analysis software) and by having the data analyzed by independent investigators who were not involved in grant application peer review.

### Strength and Weaknesses of the Study in Relation to Other Studies

The first part of our study identified assessment criteria used by both French and international funding organizations. Our typology of these criteria is consistent with previous data [5], [16], [24]. The main differences across calls for proposals related to the definitions and weighting of the criteria. For example, in French PHRCs, methodology criteria were given high weights and were described in detail and evaluated by specific questions derived in part from the CONSORT statement [25]. Differences across calls may be related to cultural factors or to the objective or scope of the calls. Further studies are needed to extend these results.

The second part of our study found evidence of heterogeneity in the review process, in keeping with previous reports [1]. Many studies assessed the level of agreement across external reviews, between internal and external reviews, and between reviews and committee decisions [6], [7], [9], [26]–[28] Agreement was usually poor. A few studies sought to identify the reasons [10], [28], [29]. Our results suggest that variability in assessment results across reviewers may be related to differences in the way reviewers conduct their assessments and use recommended criteria. **First,** we found differences across reviewers in the time spent on each review and in the review methods used, particularly regarding referral to previous studies and the use of assessment checklists. Few studies have investigated these issues [30]. One possible explanation to the differences found is the lack of formal procedures in French PHRCs. Studies of other grant organizations are needed. **Second,** internal and external reviewers differed in their perceptions of their role in the assessment process. External reviewers tended to see themselves as decision-makers. Some of the internal reviewers felt they should conduct assessments similar to those expected from external reviewers, particularly when the external reviewers failed to deliver their reports or provided reports of poor quality. Studies of perceptions have shown that external reviewers feel their role in the review process receives insufficient recognition [4], [5]. Fewer studies have investigated the viewpoints of internal reviewers [26]. As there are no formal definitions of the roles of external and internal reviewers in the French PHRC guidelines, we cannot exclude that the differences in perceptions between external and internal reviewers are specific to our sample. Additional studies are needed to explore the roles of each participant in the grant application peer review process. **Third**, we found evidence of subjectivity and heterogeneity in the way assessment criteria were used in practice by reviewers and committees. For example, reviewers seemed to prefer subjective domains, such as originality or relevance of the study, over more objective domains such as methodology. This finding contrasts with the growing emphasis placed on evidence-based medicine and with the importance given to methodology items in assessment checklists. Feasibility was also often perceived by reviewers to be an important assessment item. Feasibility was assessed objectively, based on methodological or financial considerations or, more often, subjectively, based on the reviewers’ personal experience and perceptions of the applicant’s environment. That subjective factors influence peer reviews has been suggested previously [16], [31], [32]. Subjectivity may cause two main problems: reviewers may prefer projects embedded along conventional avenues of research over innovative proposals [33], [34], and they may prefer senior researchers who already have a strong reputation over younger researchers. This last bias, known as the St Matthew paradox [1], [16], results in funds being preferentially allocated to researchers whose have received funding in the past and therefore disadvantages innovative proposals written by new researchers [33].

### Potential Implications for Policymakers and Future Research

The existence of heterogeneity in grant application assessments by reviewers may be inherent to peer review [35] and may challenge the validity of this method of grant assessment [1], [6]. However, the impact of inter-reviewer heterogeneity on the quality and effectiveness of grant application reviews [2], [36]–[40] has rarely been investigated. Several strategies might help to reduce this heterogeneity. The first strategy consists in improving the transparency of the review process [41]. International organizations such as the NIH (National Institutes of Health, US), MRC (Medical Research Council, UK), and NHMRC (National Health and Medical Research Council, Australia) have implemented transparency procedures, and a few of them allow applicants to provide answers to reviewers’ comments during the review process. Transparency could also be improved by providing applicants with transcripts of the committee discussions or by using open peer review [42]. Another strategy involves harmonizing the review procedures by developing common guidelines similar to the International Committee of Medical Journal Editors’ (ICMJE) recommendations for manuscript peer review [3], [4]. These guidelines should include definitions of the assessment criteria designed to facilitate the reviewers’ work [4] and to minimize the influence of subjective factors. Our typology constitutes a first step toward the development of such recommendations. Global standardization of all assessment and scoring procedures might, however, prove difficult to implement, given that each country and each call for proposals exhibits specific features. Uniform requirements for assessment criteria should also include clear guidance on the roles, qualifications, and duties of each participant in the review process (internal reviewers, external reviewers, and committee members). We believe that internal reviewers should act as super experts and not as additional external reviewers and that their opinion should be based on the external reviewers’ reports. Guidelines for grant application reviewers should define the reviewers’ qualifications. For example, experts specialized in financial and methodological issues could review these two aspects. Clear guidance should be provided about the course of action in the event of disagreement among reviewers of a same application. Furthermore, the reviewers should be required to substantiate their opinions. There is a need for measures designed to improve the quality of grant application reviews similar to those used to improve manuscript reviews [2], [43]–[45]. For instance, reviewer training might be helpful [46], [47], although a previous trial on manuscript review has found reviewers training not effective [48]. The grant application review process relies heavily on reviewers, who usually work on a volunteer basis free of charge. However, previous studies have shown that an increasing number of potential reviewers decline to review grant applications [4], [5], [49]. Studies are warranted to evaluate the effectiveness, feasibility, and acceptability of incentives such as financial compensation or academic recognition [50].

### Conclusion

Peer review plays a pivotal role in the selection of research proposals for funding and therefore in the nature of the scientific data produced by research. We identified a number of assessment criteria recommended by both French and international funding organizations but we also found considerable heterogeneity among the practices of reviewers. The impact of this heterogeneity on the quality and effectiveness of the review process remains unknown. Further studies are needed to investigate this issue and to develop uniform requirements for evaluating grant applications.

## Supporting Information

Appendix S1 (DOC)
