# Supplementary material for: Peer Review of Grant Applications: Criteria Used and Qualitative Study of Reviewer Practices
Source: PLoS One. 2012 Sep 28;7(9):e46054. doi: 10.1371/journal.pone.0046054 (PMC3460995; doi:10.1371/journal.pone.0046054)
Supplement: Appendix S1 — (DOC) [file pone.0046054.s001.doc]

**Appendix S1**

| **External reviewers** | **Sex** | **Age** | **Geographic area** | **Job title** | **Specialty** |
| --- | --- | --- | --- | --- | --- |
| 1 | Male | 30-39 | Paris area | Senior university-hospital physician | Psychiatry |
| 3 | Male | 40-49 | Paris area | Senior university-hospital physician | Biology |
| 8 | Male | 50-59 | Paris area | Senior university-hospital physician | Anesthesia |
| 9 | Male | 40-49 | Other region | Senior university-hospital physician | Medicine |
| 10 | Female | 40-49 | Other region | Senior university-hospital physician | Biology |
| 11 | Female | 50-59 | Other region | Senior university-hospital physician | Biology |
| 12 | Male | 50-59 | Other region | Senior university-hospital physician | Biology |
| 14 | Male | 50-59 | Other region | Senior university-hospital physician | Anesthesia |
| 15 | Male | 50-59 | Other region | Senior university-hospital physician | Medicine |
| 16 | Male | 50-59 | Paris area | Senior university-hospital physician | Medicine |
| 17 | Male | 40-49 | Paris area | Senior university-hospital physician | Surgery |
| 18 | Male | 60-70 | Other region | Senior university-hospital physician | Dentistry |
| 20 | Male | 50-59 | Paris area | Senior university-hospital physician | Biology |
| 22 | Male | 60-70 | Other region | Senior university-hospital physician | Medicine |
| 23 | Female | 50-59 | Paris area | Senior university-hospital physician | Medicine |
| 24 | Male | 50-59 | Other region | Senior university-hospital physician | Medicine |
| 25 | Female | 50-59 | Other region | Senior university-hospital physician | Medicine |
| 26 | Male | 40-49 | Other region | Senior university-hospital physician | Ophthalmology |

|  |  |  |  |  |  |
| --- | --- | --- | --- | --- | --- |
| **Internal reviewers** | **Sex** | **Age** | **Geographic area** | **Job title** | **Specialty** |
| 1 | Male | 40-49 | Paris area | Senior university-hospital physician | Biology |
| 2 | Male | 50-59 | Other region | Senior university-hospital physician | Medicine |
| 3 | Male | 40-49 | Paris area | Senior university-hospital physician | Medicine |
| 5 | Male | 40-49 | Paris area | Senior university-hospital physician | Medicine |
| 7 | Male | 50-59 | Other region | Senior university-hospital physician | Methodology |
| 10 | Female | 50-59 | Paris area | Physician not working in a university hospital | Methodology |
| 11 | Male | 40-49 | Paris area | Senior university-hospital physician | Biology |
| 14 | Male | 40-49 | Paris area | Senior university-hospital physician | Medicine |
| 16 | Male | 40-49 | Paris area | Senior university-hospital physician | Methodology |
| 17 | Male | 60-69 | Paris area | Senior university-hospital physician | Surgery |
| 18 | Male | 40-49 | Paris area | Senior university-hospital physician | Biology |
| 19 | Male | 40-49 | Paris area | Senior university-hospital physician | Gynecology and Obstetric |
| 20 | Male | 40-49 | Paris area | Senior university-hospital physician | Biology |
| 25 | Male | 60-69 | Paris area | Senior university-hospital physician | Medicine |
| 26 | Male | Unknown | Other region | Senior university-hospital physician | Medicine |
| 30 | Male | 50-59 | Paris area | Senior university-hospital physician | Medicine |
| 33 | Female | 40-49 | Paris area | Senior university-hospital physician | Methodology |
